# First Immunohistochemical Demonstration of the Expression of a Type-2 Vomeronasal Receptor, V2R2, in Wild Canids

**DOI:** 10.3390/ijms25137291

**Published:** 2024-07-02

**Authors:** Irene Ortiz-Leal, Mateo V. Torres, Ana López-Beceiro, Luis Fidalgo, Taekyun Shin, Pablo Sanchez-Quinteiro

**Affiliations:** 1 Department of Anatomy, Animal Production and Clinical Veterinary Sciences, Faculty of Veterinary, University of Santiago de Compostela, Av. Carballo Calero s/n, 27002 Lugo, Spain; irene.ortiz.leal@usc.es (I.O.-L.); mateovazquez.torres@usc.es (M.V.T.); anam.lopez.beceiro@usc.es (A.L.-B.); luis.fidalgo@usc.es (L.F.); 2 College of Veterinary Medicine and Veterinary Medical Research Institute, Jeju National University, Jeju 63243, Republic of Korea; shint@jejunu.ac.kr

**Keywords:** vomeronasal organ, V2R, G-proteins, Canidae, immunohistochemistry

## Abstract

The mammalian vomeronasal system enables the perception of chemical signals crucial for social communication via the receptor families V1R and V2R. These receptors are linked with the G-protein subunits, Gαi2 and Gαo, respectively. Exploring the evolutionary pathways of V1Rs and V2Rs across mammalian species remains a significant challenge, particularly when comparing genomic data with emerging immunohistochemical evidence. Recent studies have revealed the expression of Gαo in the vomeronasal neuroepithelium of wild canids, including wolves and foxes, contradicting predictions based on current genomic annotations. Our study provides detailed immunohistochemical evidence, mapping the expression of V2R receptors in the vomeronasal sensory epithelium, focusing particularly on wild canids, specifically wolves and foxes. An additional objective involves contrasting these findings with those from domestic species like dogs to highlight the evolutionary impacts of domestication on sensory systems. The employment of a specific antibody raised against the mouse V2R2, a member of the C-family of vomeronasal receptors, V2Rs, has confirmed the presence of V2R2-immunoreactivity (V2R2-ir) in the fox and wolf, but it has revealed the lack of expression in the dog. This may reflect the impact of domestication on the regression of the VNS in this species, in contrast to their wild counterparts, and it underscores the effects of artificial selection on sensory functions. Thus, these findings suggest a more refined chemical detection capability in wild species.

## 1. Introduction

The ability of organisms to adapt to their environment is crucial for the survival of both individual members and the species as a whole. Evolutionary processes have endowed mammals with complex sensory systems, which are specialized organs crucial for sensing environmental cues and enabling continuous adaptation [1,2]. Among these, the chemical senses, particularly olfactory subsystems, are key to managing essential survival functions, including communication among members [3], social interactions [4,5], and mating behaviors [6,7]. Chemical communication between individuals depends on chemical signals called semiochemicals [8,9], which are produced by individuals, released into the environment through secretions [10,11], and recognized by the olfactory systems of animals [12,13].

The principal mechanisms for odor and pheromone detection comprise the main olfactory system (MOS) and the vomeronasal system (VNS). The former operates through the main olfactory epithelium and the olfactory bulb and has a significant linkage to the limbic system, playing the main role in both memory retrieval and conscious perception [14]. On the other hand, the VNS, which comprises the vomeronasal organ (VNO) and the accessory olfactory bulb (AOB), is implicated in non-conscious influences in sexual behavior [15,16], maternal identification [17,18], and the detection of predators [19,20], and, therefore, it is specifically tuned to sensing of pheromones [21,22], kairomones [12], steroids [23,24], and major histocompatibility complex (MHC) molecules [25,26].

Despite their proximity, these two systems maintain anatomical independence, exhibiting significant morphological and functional disparities, which suggest that they likely evolved separately [27,28,29,30]. However, functional dichotomy has been questioned, which is based on the presence of vomeronasal receptors (VR) in the olfactory mucosa and odorant receptors in the VNO [31,32,33].

Rodent species represent the primary model for studies concerning the VNS in mammals [34,35,36,37,38]. Shinohara and colleagues [39] first revealed the presence of Gαi2 and Gαo into non-overlapping regions of the AOB, showing that the rostral part expressed Gαi2, whereas the caudal one expressed Gαo. Since the vomeronasal sensory neurons (VSNs) send their axonal projections to the AOB, it was possible to demonstrate that also the VNO is provided with a similar anatomical organization in several rodent species, in that Gαi2 is specifically expressed in the apical neurons of the VNO, whereas Gαo is expressed in the basal ones [40,41,42]. Unraveling the involvement of both G-proteins within the signal transduction sequence in VSNs proved essential to the discovery of the two main VR families. In fact, in the mouse VNO, Dulac and Axel [43] identified a set of receptors, named V1Rs, whose expression pattern specifically matched the Gαi2 expression profile.

The identification of a second VR family, type-2 V2R, in rat and mouse occurred in 1997 through simultaneous studies [44,45,46], cementing the notion of dual independent sensory pathways. In fact, V2Rs were shown to exclusively co-express with Gαo in the basal neurons of the VNO. Thus, the immunohistochemical profiling of Gαi2 and Gαo since has become a key method for indicating V1R and V2R receptor expression in the VNO, respectively. However, later studies, in the VNO of other mammals, such as goat [47], dog [48,49], horse and marmoset [48], sheep [50], hyrax [51], cat [52], hippopotami [53], meerkat [54], and cow and pig [55], demonstrated the expression of Gαi2 but not of Gαo. Unexpectedly, the wallaby (genus *Notamacropus*) was initially proposed as an alternative model in the organization of G-protein expression, only showing Gαo immunostaining, which was localized in the basal neurons of the VNO. Unexpectedly, it was scattered throughout the AOB without any evident compartmentalization [56]. However, it has recently been demonstrated that this marsupial genus possesses canonical immunohistochemical labeling of Gαo and Gαi2 in both VNO and AOB [57].

The differentiation of two segregated pathways for pheromonal information processing, as demonstrated by immunohistochemistry, has received further support from genomic studies in different species [58,59,60,61]. However, more recent immunohistochemical observations have revealed the expression of Gαo in the VNO of species that, based on the available genomic data, were assumed to lack V2Rs, specifically in wild canids such as the fox and wolf [62,63], suggesting caution when dealing with incompletely annotated genomes, as is often the case of wild species.

In recent years, evidence has emerged from genomic studies suggesting that this dichotomy might need to be revisited. Kondoh et al. [55] in their whole-genomic studies of VR receptors in artiodactyls determined the presence in cows, pigs, sheep, and goats of a significant number of functional V2R genes. Moreover, Hohenbrink et al. [64] identified for the first time two intact V2R loci in a strepsirrhine primate, which until then were assumed to lack them. In light of these results, caution is advised when interpreting genomic results in a phenotypic context and even more so when dealing with incompletely annotated genomes, as is often the case in wild Canidae. Given that genomic datasets in wild Canidae are typically constructed from a limited number of individuals, it becomes imperative to approach these data with greater care. A higher number of sequenced individuals not only enhance understanding of genetic diversity but also improve the accuracy and robustness of results, aiding in accurately inferring population structure.

In this context, a phenotypic characterization of the expression of vomeronasal receptors, especially of V1Rs and V2Rs in the VNO, would be desirable. For this reason, we have characterized the expression, through an immunohistochemical approach, of V2Rs in the VNO of wolf and fox, in which Gαo is abundantly expressed [62,63]. In this immunohistochemical study, we have also included the VNO of the dog since, in this species, there is no definitive information to date [49,65].

## 2. Results

In our study, we have applied immunohistochemical techniques to investigate the expression pattern of V2R2 in the vomeronasal epithelium of three canid species: two wild, the wolf and the fox, and one domestic, the dog.

We have taken advantage of the availability of anti-V2R2, which is an antibody that recognizes all functional receptors of family-C V2Rs [66]. Anti-V2R2 is a polyclonal antibody that has been exhaustively characterized and validated in the mouse and rat VNO [67,68]. We used this antibody, as family-C V2R are highly conserved throughout species.

**Figure 1:**
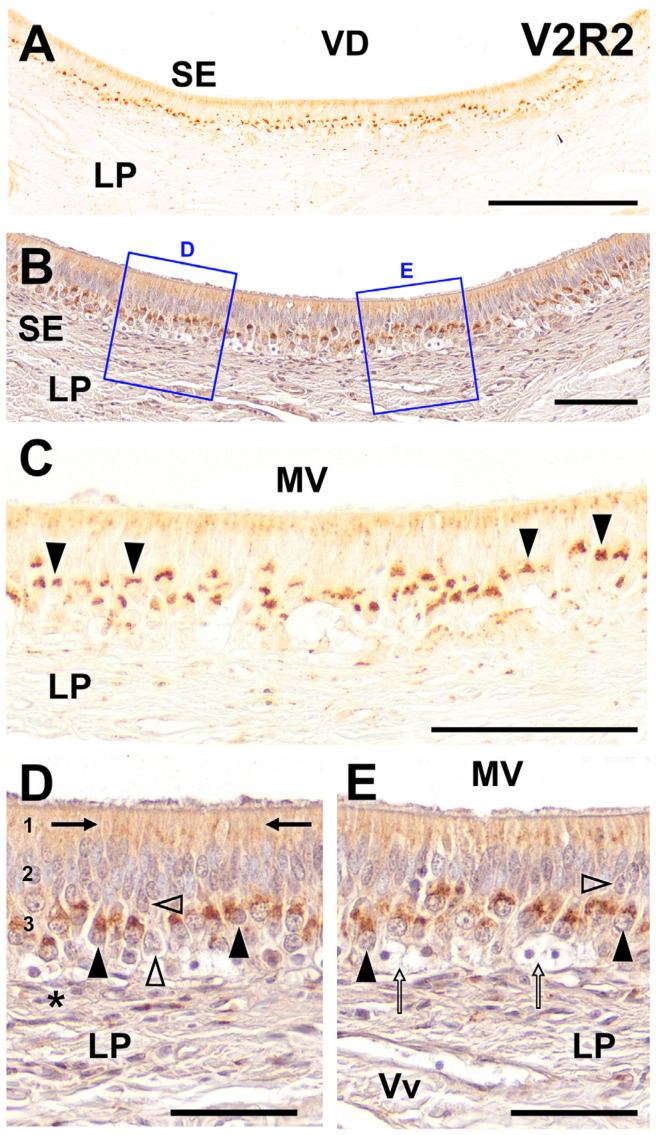
Immunohistochemical labeling of the wolf VNO using anti-V2R2 antibody. (**A**) Labeling is observed throughout the entire strip of the vomeronasal sensory neuroepithelium (SE), particularly in its basal part. Additionally, the apical portion of the epithelium exhibits a more diffuse labeling. (**B**) Counterstaining with hematoxylin of the neuroepithelium confirms this labeling pattern and allows for the identification of the structural characteristics of the cellular strata and lamina propria (LP). (**C**) At higher magnification, the labeling is observed to correspond to cells that, because of their spatial placement and morphological characteristics, are identified as neuroreceptor cells (arrowheads). This labeling exhibits a clean and well-defined pattern that is predominantly localized to the soma. In addition, a more diffuse pattern of labeling is evident in the apical region of the epithelium. (**D**,**E**) Higher magnification of the counterstained areas shown in 1B allows for the determination of the morphological features of the immunopositive structures. It is noticeable that in the immunopositive cells (black arrowhead), the labeling is concentrated in the apical portion of the somas. From this portion, dendrites emerge and project toward the apical surface to form the immunopositive dendritic processes (arrows). Not all receptor cells are immunopositive for V2R2, with cellular somas in different locations being immunonegative (open arrowheads). Finally, no immunopositivity is observed in the various basal cells (asterisk and open arrows). (1) Apical processes; (2) Sustentacular cells layer; (3) Neuroreceptor cells layer. MV, mucomicrovillar complex; VD, vomeronasal duct; Vv, blood vessels. Scale bars: (**A**): 250 μm; (**B**–**E**): 100 μm.

The results of the immunolabeling with anti-V2R2 in the wolf VNO are presented in Figure 1. The analysis revealed extensive labeling throughout the entire extent of the vomeronasal neuroepithelium with pronounced intensity in its basal part. Moreover, the apical portion of the epithelium displayed a more diffuse labeling in the dendritic processes of the VSNs. This pattern suggests a significant expression of V2R2 in the wolf VNO (Figure 1A).

Counterstaining with hematoxylin further confirmed the observed labeling pattern and facilitated the delineation of the structural characteristics of the cellular strata and the underlying lamina propria as well as the localization of V2R2 within specific cellular compartments (Figure 1B). A higher magnification of both non-counterstained (Figure 1C) and counterstained (Figure 1D,E) images provided a clearer insight into the cellular specificity of the V2R2 expression. The labeling corresponded predominantly to cells identified as VSNs based on their spatial placement and distinctive morphological features. These cells exhibited a clean and sharply defined pattern of labeling that was primarily concentrated in the soma. Additionally, we observed a diffuse labeling pattern in the apical region of the epithelium (Figure 1C). The counterstained slides showed that the labeling in the VSNs was concentrated in the apical portion of their somata (Figure 1D). Dendrites originating from these somata extended toward the apical surface, culminating in the formation of immunopositive dendritic processes (Figure 1D).

Interestingly, not all the receptor cells displayed immunopositivity for V2R2 with some cellular somata in various locations remaining unstained (Figure 1D,E). Additionally, our observations confirmed the absence of immunopositivity in the diverse basal stem cells (Figure 1E). These findings underscore the specific localization and expression patterns of V2R2 within the wolf VNO, highlighting the receptor potential role in pheromonal signaling and its implications in the sensory functions of this species.

**Figure 2:**
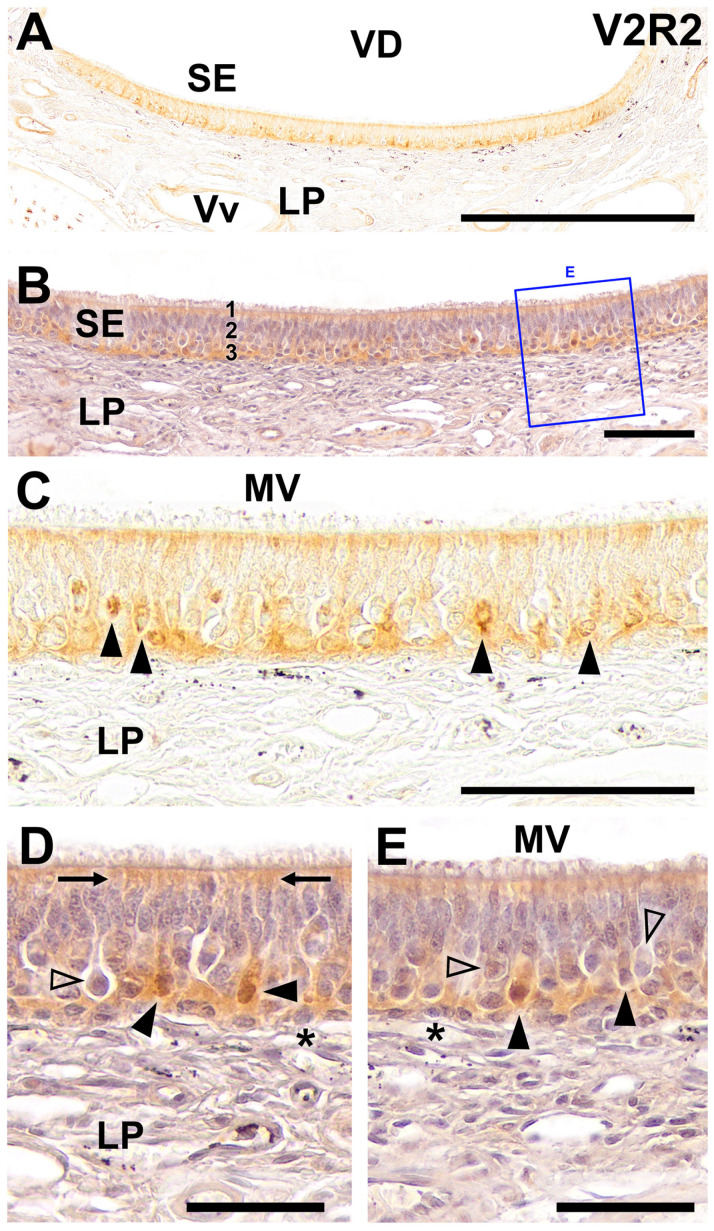
Immunohistochemical labeling of the fox VNO using anti-V2R2 antibody. (**A**) Throughout the vomeronasal sensory neuroepithelium (SE), consistent labeling is evident, which is especially pronounced in the basal area. Additionally, the apical zone shows a diffuse pattern of labeling. (**B**) Hematoxylin counterstaining of the neuroepithelium facilitates the visualization of the structural features of the cellular layers and lamina propria (LP). (**C**) Higher magnification corroborates the established labeling pattern and also enables detailed recognition of the cellular strata and the underlying lamina propria. This labeling displays a distinct arrangement that is mainly concentrated in the soma (arrowheads). Additionally, the apical part of the epithelium exhibits a more widespread staining pattern. (**D**,**E**) Detailed imaging of the counterstained SE, including the section shown in 2B, permits the assessment of the morphological features of the immunopositive structures. The immunopositive cells (black arrowheads) have labeling concentrated at the somata apical end. The dendritic ends are as well immunopositive (arrows). Not every vomeronasal neuron is immunopositive for V2R2 with some cell bodies in different locations showing immunonegativity (open arrowheads). The basal cells display no immunopositivity (asterisk). Scale bars: (**A**): 500 μm; (**B**–**E**): 100 μm.

In the immunohistochemical study of V2R2 expression in the sensory epithelium of the fox VNO, we have as well obtained consistent labeling throughout the vomeronasal sensory neuroepithelium, which was especially pronounced in the basal area (Figure 2). Additionally, the apical zone shows a diffuse pattern of labeling (Figure 2A). Hematoxylin counterstaining of the neuroepithelium facilitates the visualization of the structural features of the cellular layers and the lamina propria (Figure 2B). No counterstained higher magnification images confirm the established labeling pattern. This labeling displays a distinct arrangement that is mainly concentrated in the soma. Additionally, the apical part of the epithelium exhibits a more widespread staining pattern (Figure 2C). Counterstaining at higher magnification shows the labeling mostly concentrated at the somata apical end. The dendritic ends are also immunopositive. As expected, not every VSN cell is immunopositive for V2R2. The basal stem cells display no immunopositivity (Figure 2D,E). These findings highlight the expression of V2R2 within the VNO sensory epithelium of the fox.

**Figure 3:**
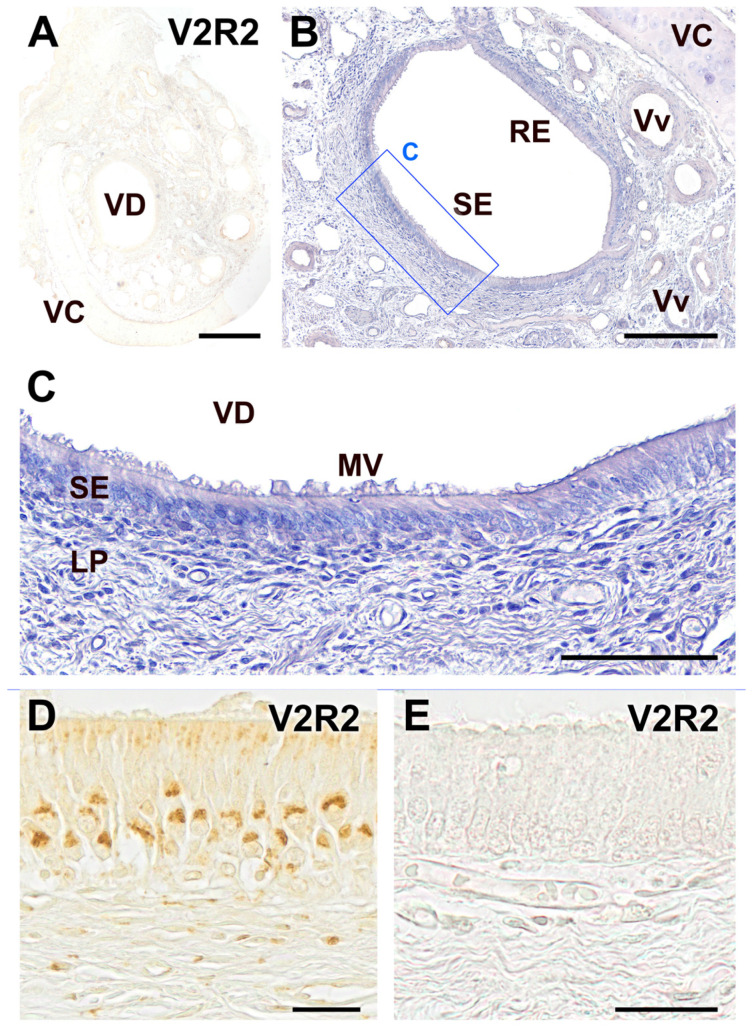
Immunohistochemical labeling of the dog VNO using anti-V2R2 antibody. (**A**) The immunohistochemical study of the dog VNO using antibodies against V2R2 did not produce any immunostaining in any of the structural components that constitute it. An immunonegative section is shown. (**B**) The same section hematoxylin counterstained reveals the differentiation between the sensory epithelium (SE) and the non-sensory or respiratory epithelium (RE). (**C**) An image of the SE at higher magnifications, in which the neuroepithelium cellular strata, the mucomicrovillar complex, and the underlying lamina propria can be seen, all of them immunonegative. (**D**,**E**) High-magnification images of the VNO sensory epithelium. Comparison of the wolf anti-V2R2 immunopositive labeling (**D**), with the dog immunolabeling (**E**), which produces a complete lack of immunostaining. Scale bars: (**A**): 500 μm; (**B**): 250 μm; (**C**): 100 μm; (**D**,**E**): 25 μm.

The immunohistochemical labeling of the dog VNO using the anti-V2R2 antibody did not result in immunostaining of any structural components of the organ in all the examined sections (Figure 3A,E), which contrasts with the clear labeling obtained in the vomeronasal sensory epithelium of the wolf (Figure 3D). Counterstaining of the immunostained sections allowed for the clear distinction between the sensory epithelium and the non-sensory or respiratory epithelium, highlighting the structural organization of the dog VNO (Figure 3B,C). The sensory epithelium displayed the neuronal layers, the mucomicrovillar complex on its surface, and the underlying lamina propria. All of these were found to be immunonegative, confirming the absence of V2R2 immunoreactivity in these structures (Figure 3E).

**Figure 4:**
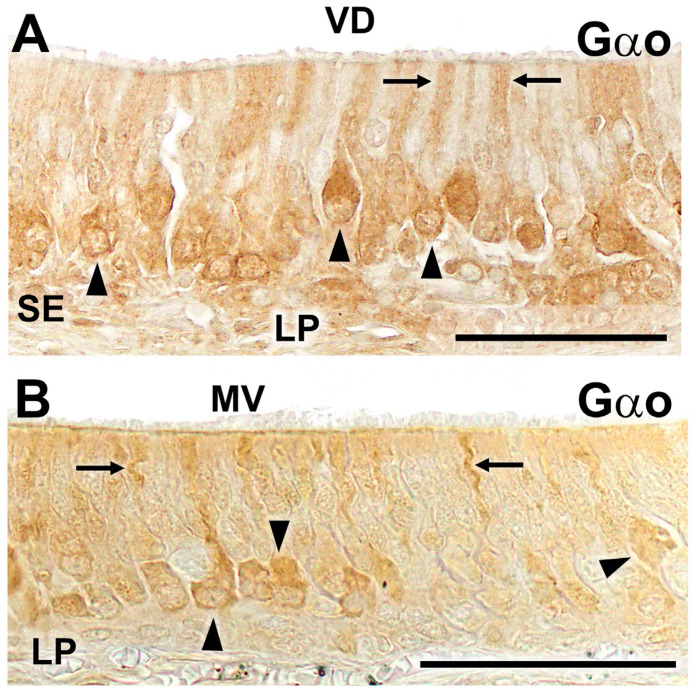
Immunohistochemical labeling of the wolf and fox VNO using anti-Gαo antibody. (**A**) Immunohistochemical labeling against Gαo in the sensory epithelium of the wolf VNO results in positive staining in a subpopulation of sensory neurons, which are predominantly located in the basal position. The labeling concentrates on the cellular somata (arrowheads) and the dendritic processes (arrows). (**B**) The study with the same antibody in the fox VNO produces a similar staining pattern with immunopositive cellular somata (arrowheads) and dendrites (arrows). Scale bars: (**A**,**B**): 50 μm.

Finally, given that the expression of V2Rs is tightly linked to that of Gαo, we conducted an immunohistochemical study on VNO sections of the wolf and fox vomeronasal organ to assess whether this transduction protein was also detectable in these samples. The results are presented in Figure 4. In both species, immunopositivity for Gαo was observed in the sensory epithelium of the VNO. The immunolabeling comprised a subpopulation of non-receptor cells and extended to both the cellular somas and the dendritic processes present on the luminal surface (Figure 4A,B).

Table 1 highlights the species-specific differences in the expression patterns of V2R2 and Gαo in the vomeronasal organ, reflecting possible variations in pheromonal detection capabilities influenced by domestication and species-specific sensory evolution.

## 3. Discussion

The vomeronasal system in mammals has undergone highly sophisticated and complex evolution, leading to significant diversity within vomeronasal receptor families that are characterized by extensive gene diversity and gene inactivation across species [29,69]. Understanding how this diversity arose requires a phylogenetic perspective, in which the system is characterized from a morphological, biochemical, and comparative genomic viewpoint, avoiding making extrapolations between groups, even those that appear to be very closely related, and taking into account the interaction between the main and accessory olfactory systems [30,70].

For decades, it has been widely accepted that there are two models of expression for the two main families of vomeronasal receptors, V1R and V2R: a segregated model (expressing both families) and a uniform model (expressing only V1R). The segregated model would include rodents [71,72], lagomorphs [73], marsupials [40], and strepsirrhine primates [64,74], while the rest of the investigated mammals would fall under the uniform model [75,76]. The distinction between these models was based on information originally derived from the expression of the Gαi2 and Gαo, as they are considered relevant markers for the expression of V1Rs and V2Rs, respectively. The development of genomics in recent decades has helped to clarify the enormous disparity and diversity of morphological data available on the vomeronasal system. However, there are still some mismatches between genetic and morphological information in numerous mammalian groups [77,78]. Current genomic evidence sometimes clashes with immunohistochemical data and vice versa as immunological features challenge the information provided by genomics.

In our study of the vomeronasal system in wild canids, specifically wolves and foxes, we encountered the latter situation. While the current genomic information seems to exclude the presence of V2Rs in these species, thus assigning them to the uniform model, our immunohistochemical data demonstrate the expression of both Gαo and V2Rs at the protein level, thereby aligning the VNO of fox and wolf with the segregated model.

To our knowledge, this study represents the first demonstration of the expression of a V2R beyond a laboratory rodent model, namely rat and mouse. This has also allowed the confirmation of the likely role in pheromone transduction of Gαo-positive cells found in both species, fox and wolf. As V2R2 belongs to family-C V2Rs, this finding has additional implications. Studies on the expression of V2R2 have demonstrated that genes of family-C are expressed in all basal neurons of the VNO and are highly conserved throughout species, making its antibody useful for detecting Gαo pathways in many mammals.

Sequence comparison demonstrates that V2R2 is a divergent member of the V2R family. This divergence, however, does not detract from its sensory nature. Previous studies suggest that despite its unique sequence characteristics, V2R2 retains functional roles similar to other V2Rs, contributing to the diversity and complexity of vomeronasal sensory perception [68]. The expression of V2R2 in mice and rats is restricted to the VNO and specifically to the Gαo-expressing cells within this neurosensory epithelium [45,46]. Double-labeling experiments have clearly demonstrated the co-expression of different V2Rs in V2R2-containing sensory neurons. This widespread expression suggests a fundamental role for V2R2 in vomeronasal function [66].

Although the expression of two vomeronasal receptors in the same neuroreceptor cell may appear to violate the established rule of ‘one receptor per neuron’ in the olfactory system, growing evidence suggests that this principle might not be universally applicable [79,80,81]. Moreover, the V2R-related family of receptors in fish olfactory epithelia displays a pattern of varied expression among neurons. Specifically, it has been demonstrated that two V2R-related goldfish receptors, 5.24 and 5.3, are expressed in a large subset of olfactory neurons. This indicates that receptors of this family are likely co-expressed in the same cells [82].

The hypotheses about the functional role of V2R2 receptors are varied and insightful. For instance, it has been proposed that amino acids may bind to V2R2, suggesting its involvement in detecting molecules with α-carboxyl-amino groups, which could be crucial for pheromonal communication [67]. Another approach suggests that the co-expression of V2R2 with other V2R receptors might be implied in alternative modes of chemosensory information processing. For example, ligands for V2R2 could modify the responses mediated by other V2Rs, either sensitizing or desensitizing the cells to various stimuli. This interaction could play a significant role in the overall sensory perception mechanism of the VNO [66]. Furthermore, an alternative hypothesis is that V2R2 might form heterodimers with other V2Rs. For example, it has been shown that GABA-b receptors are composed of two distinct but related G-protein-coupled receptors [83,84].

In the case of wolves and foxes, we lack additional antibodies against V2R family receptors to confirm their co-expression with V2R2 receptors. However, the extensive pattern of V2R2 expression that we have observed closely mirrors that seen in rodent families, suggesting a strong likelihood of V2R2 co-expression within the vomeronasal neuroepithelium of wolves and foxes. Future research will be crucial in conclusively determining these co-expression patterns.

The results obtained in the current study regarding immunostaining with V2R2 in the VNO of both wolf and fox are clearly consistent with the expression of this receptor in the vomeronasal neuroepithelium. Specifically, the staining is clear both in the somas and in the dendritic processes that reach the mucomicrovillar complex covering the luminal surface of the epithelium. It is noteworthy that there is no staining in the axonal terminals that leave the somas at their basal part with staining concentrated on the apical part of the somas. This absence of axonal staining coincides with observations made by Martini and colleagues [66] in mouse and rat.

Although previous studies on the expression of Gαo in the VNO of fox and wolf were already available [62,63], for this study, we have confirmed these results using antibodies against Gαo from two different commercial brands. In both cases, the staining pattern is comparable to that observed with the V2R2 antibody. It is noteworthy that while in the case of laboratory rodents, there is a basal–apical zonation of the VNO neuroepithelium in terms of G-protein and receptor expression, in canids, the reduced size of the organ does not allow for highlighting this differentiation. Our observations of a high proportion of V2R2-positive cells in the wolf VNO align with the detected expression of the Gαo protein in the same organ. It is remarkable that species with a VNS segregated model, expressing both V1R and V2R, such as rats, mice, and rabbits, show a high proportion of V2R [41]. Determining the exact proportion of V2R and V1R cells across the entire vomeronasal organ of the wolf would be valuable, but such a study must take into account the potential variability in the organization of the neuroepithelium along the rostrocaudal axis of the VNO. Additionally, the morphometric study would require a significant number of animals in good condition, which is challenging to obtain from this wild species. We aim to conduct this morphometric analysis in future work to further elucidate these dynamics.

A second notable aspect of our study is the absence of immunoreactivity for V2R2 in the VNO of the domestic dog. This finding, however, aligns with the accepted absence of Gαo expression found in the vomeronasal epithelium of this species [49]. The contrast in the expression patterns of Gαo and V2R2 between dogs and the closely related wild canids, like wolves and foxes, offers valuable evidence that complements existing morphological analyses in the VNS of these species [62,63,85,86], which show remarkable differences in the degree of differentiation of both the VNO and the AOB. These features may suggest a regression of the system due to the effects of domestication. This issue has also been postulated in the study of the main olfactory system of the dog through different approaches [87,88,89]; therefore, our study may impact the understanding of some of the effects of domestication.

Considering the differences in sensory capabilities between domestic dogs and wolves, the significance of V2R receptors in wolf behavior becomes apparent. Scent communication, crucial for the wolf social structure [90,91], underscores the importance of these receptors. The role of V2R receptors in social behaviors, such as male aggression, has been well documented by Chamero et al. [35]. Their study emphasizes that Gαo is essential for the neural coding of chemosensory cues that modulate intermale aggressive interactions. Additionally, the critical function of V2R receptors in sex discrimination has been elucidated [92], underscoring their significance in chemically-based communication aspects, such as mating behavior. It remains surprising that a family of V2R receptors, playing a fundamental role in processing complex social and individual information, may have been lost.

Finally, regarding the discrepancy between the available genomic information on the absence of V2R receptors in the genome of canids and the phenotypic results provided by G proteins, a probable explanation might lie in the absence of exhaustively annotated complete genomes in species such as the fox and the wolf. It is feasible that genomic studies may be overlooking the presence of V2R receptors. For example, in the recent attempt to sequence and assemble the red fox genome, intact V2R genes were not reported [93]. Multiple factors, such as the quality of sequencing, the potential functionality of pseudogenes [94], and the experimental validation of findings, must be considered before definitively concluding from the genomic dataset the absence of any relevant function of V2R genes in canids. It is crucial to ensure that the coverage and quality of V2R gene sequencing are high. Some genomic regions can be challenging to sequence due to their complex structure, which could lead to an incomplete or erroneous representation of V2R genes. Additionally, if canids exhibit a unique pattern of pseudogenization or absence of certain sequences, this could indicate specific evolutionary adaptations rather than a complete loss of function. Moreover, the presence of highly pseudogenized sequences in canines and other mammals suggests that these genes have undergone complex evolutionary processes that might include both the loss and gain of functions over time. These facts underscore the importance of further genomic exploration and annotation in these species to fully understand the scope of V2R receptor expression and functionality.

Our results highlight the necessity to identify the specific projection site within the olfactory bulb for neuroreceptor cells that express the V2R2 receptor and therefore the Gαo protein. Although previous immunohistochemical studies in the accessory olfactory bulb of foxes and wolves have not found the expression of Gαo [63,85], the investigation in the fox of the transitional zone between the main olfactory and accessory olfactory bulbs, known as the olfactory limbus (OL), has identified the presence of a glomerular territory, which is remarkable for its morphology and neurochemical pattern as well as distinctly different from those of the surrounding main olfactory bulb (MOB) and accessory olfactory bulb (AOB) [86]. Significantly, this olfactory limbus exhibits in the fox an uncommonly high degree of development and complexity, suggesting a sophisticated mechanism for sensory integration. It suggests that the fox OL may be involved in the processing of specific stimuli signaling-relevant intraspecific socio-sexual cues, which is similar to the suggested functionality of this region in laboratory rodents [95,96,97]. To date, there is no comparable information in the olfactory limbus of wolves. Future studies focusing on the wolf olfactory bulb should contribute to clarifying this aspect in these species.

In conclusion, this study reinforces the existence of a segregated model in the development of the VNS in wild canids with the expression of the two vomeronasal receptor families, V1R and V2R, in species previously thought to exhibit deterioration of the latter. Additionally, it supports the notion that domestication may have produced a dramatic effect on the capacity of detecting pheromonal information.

## 4. Specific Conclusions and Future Perspectives

Our investigation into the vomeronasal system of wild canids, specifically wolves and foxes, reveals an unexpected alignment with the segregated model of vomeronasal receptor expression, contradicting the current genomic data which places these species under a uniform model. This discovery not only highlights the complex evolutionary trajectory of sensory systems in mammals but also emphasizes the potential discrepancies between genomic predictions and protein expression. The presence of V2R2 and its co-expression with Gαo in the neuroepithelial cells of the VNO in wolves and foxes suggests a more sophisticated chemical communication system than previously recognized in these species. This supports the hypothesis that wild canids retain a high capacity for pheromonal detection—a trait potentially diminished in domesticated dogs.

### Future Research Directions

Co-expression studies: Co-expression studies should be expanded to include double confocal immunohistochemical analyses to verify whether other V2R family receptors are co-expressed with V2R2 in wolves and foxes. This could require the development of new antibodies specifically raised for these receptors. Additionally, such studies would enable a comprehensive characterization of the neurochemical properties of V2R2 neuroreceptor cells, providing deeper insights into their functional roles within the sensory pathways of these species.

Genomic reanalysis: Given the findings, a reevaluation of the genomic data for canids is justified to detect possibly overlooked or misannotated V2R genes.

Functional analysis: Experimental studies are needed to explore the functional implications of V2R2 in the sensory perception of wolves and foxes, which could involve behavioral assays to understand the ecological and social roles of these receptors.

Comparative studies: Expanding the study to include more wild canid species and other carnivores would enable assessing the conservation or divergence of vomeronasal receptor systems, providing deeper insights into their evolutionary paths and functional adaptations.

Morphometric studies: It is also important to quantitatively assess the differences between species and characterize potential variations in the expression of V2R2 receptors along the entire length of the vomeronasal duct. These studies will provide a detailed analysis of the spatial distribution and density of V2R2 receptors, offering insights into how expression patterns may vary between species and across different regions of the VNO.

## 5. Strengths and Limitations of the Study

Our study provides new insights, representing the first immunohistochemical demonstration of V2R expression beyond the typical laboratory models and shedding light on mammalian chemosensation. We employed well-validated antibodies and comprehensive immunohistochemical techniques, ensuring the precise localization and characterization of receptor expressions. By comparing species with varying levels of domestication, our research highlighted the impact of domestication on sensory systems.

However, the study also faces certain limitations. The scope of our research was constrained by the limited availability of antibodies that can specifically bind to canid V2Rs, which might not cover all the V2R subtypes potentially present in these species. Additionally, our reliance on existing genomic databases, which may lack complete or accurate annotations for all canid species, particularly wild ones, posed further challenges. Moreover, our findings are based on a relatively small number of individuals, which may not fully represent the genetic diversity or receptor variability within the species studied. Finally, no Western blots for the V2R2 antibody in canid VNO are available.

By addressing these limitations in future research, we aim to enhance our understanding of the evolution and function of the vomeronasal system across different mammalian lineages with a particular focus on canids. This approach will not only refine the existing data but also expand our knowledge of sensory biology in a broader context.

## 6. Materials and Methods

In this research, we employed samples from three adult male wolves (*Canis lupus signatus*), three adult male foxes (*Vulpes vulpes*), and three adult male mixed-breed dogs (*Canis lupus familiaris*). The wolves were sourced from wildlife rehabilitation centers located in the Galicia provinces and had succumbed to fatal accidents. We selected only those individuals that had recently passed away and exhibited no signs of external or internal injuries to the head for inclusion in our study. The foxes came from activities organized by the Galician Hunting Federation. They were obtained in the field, the same day of their shooting, with a maximum of two-hour delay. The procurement of wolves and fox samples was conducted in compliance with the necessary authorizations from the Galician Environment, Territory, and Housing Department, under the approval codes EB-009/2020 and EB-007/2021. The dogs subjected to necropsy were adult mesocephalic canines, sourced solely from the Department of Clinical Sciences at our institution, where they had died for various clinical conditions. Their heads were intact, displaying neither clinical nor postmortem signs of neurological disorders.

Upon their arrival to the Unit of Anatomy of the Faculty of Veterinary in Lugo, all heads were promptly dissected. The VNOs were exposed by removing the nasal bones and the lateral walls of the nasal cavity. The bone tissue encasing the VNO on the ventral and medial sides was meticulously dissected away from each sample, allowing for processing without the necessity for decalcification. Subsequently, the samples were immersed in Bouin’s fluid (BF), composed of 75% saturated picric acid (Sigma P6744, St. Louis, MO, USA), 20% formaldehyde (Sigma 252549, St. Louis, MO, USA), and 5% acetic acid (Sigma 33209, St. Louis, MO, USA) for a 24 h period, then transferred to 70% ethanol (diluted from 100% ethanol Sigma 02860, St. Lois, MO, USA), embedded in paraffin (Paraplast 15159-409, Leica Microsystems GmbH, Wetzlar, Germany), and sectioned using a microtome (Leica 2055, Leica Microsystems GmbH, Wetzlar, Germany). They were systematically sectioned in a transverse plane along their entire length, from the caudal end to the rostral end, at a thickness of 6–7 µm.

### 6.1. Immunohistochemistry Methodology

After deparaffinizing and rehydrating the samples, they were processed without performing antigen retrieval. This approach was chosen to avoid unmasking epitopes that could result in non-specific binding. The first step involved treating all samples with a 3% H_2_O_2_ solution to inhibit endogenous peroxidase. Subsequently, samples were immersed in a 2.5% horse serum solution that was compatible with the ImmPRESS Anti-Rabbit IgG reagent kit (Vector Laboratories, Burlingame, CA, USA) to preclude non-specific binding. Samples were incubated overnight at 4 °C with the primary antibodies.

The next day, samples were incubated for 30 min with the ImmPRESS VR Polymer HRP Anti-Rabbit IgG (Vector Laboratories, Burlingame, CA, USA). Prior to the visualization stage, all samples were rinsed for 10 min in 0.2 M Tris-HCl buffer at pH 7.61. DAB chromogen was used for visualizing. A 0.05% 3,3′-diaminobenzidine (DAB, Sigma D7679, St. Lois, MO, USA) chromogen solution and a 0.003% H_2_O_2_ solution in 0.2 M Tris-HCl buffer were used. The DAB reagent develops into a brown precipitate in the presence of the hydrogen peroxide solution, which enables the visualization of the reaction.

Negative controls omitted the primary antibodies. As positive control, we employed sections from tissues known to express the protein of interest.

To carry out the counterstaining procedure, after taking the necessary microphotographs for the immunohistochemical study, the slides were immersed in xylene (Sigma 295884, St. Louis, MO, USA) for 24 h to facilitate the removal of the cover slips. Once this was accomplished, they were cleared and rehydrated to stain them with hematoxylin (Sigma GHS132, St. Louis, MO, USA) for 5 min. Finally, the samples were dehydrated, cleared, and mounted once more, and microphotographs were taken of them.

### 6.2. Primary Antibodies

The antibody against V2R2 was developed by Prof. Tirindelli (University of Parma, Italy). It is a polyclonal antibody raised in rabbit [68]. For the immunohistochemical study of the expression of the G protein alpha subunit Gαo, two commercial polyclonal antibodies were used. The reason for employing two different antibodies was to confirm the immunolabeling obtained with this protein, which in both cases was analogous. The antibodies used were from Santa Cruz Biotechnology (sc-387, Dallas, TX, USA) and Medical & Biological Laboratories (MBL-551; Medical & Biological Laboratories, Nagoya, Japan).

### 6.3. Image Capture

Images were digitally captured using a Karl Zeiss Axiocam MRc5 camera coupled with a Zeiss Axiophot microscope (Zeiss, Oberkochen, Germany). Adobe Photoshop CS4 (Adobe Systems Incorporated, San Jose, California, USA) was employed for brightness, contrast and balance adjustment; however, no enhancements, additions, or relocations of the image features were made. Additionally, an image-stitching software (PTGuiPro, vs.12.26, New House Internet Services B.V., Rotterdam, The Netherlands) was used for low-magnification images composed of several photographs.

## Figures and Tables

**Table 1:** Presence of V2R2-ir and Gαo-ir in the VNO of wild and domestic canines.

Species	V2R2-ir in VNO	Gαo-ir in VNO	Observations
Wolf	Positive	Positive	Extensive labeling throughout the VNO neuroepithelium. V2R2 expression is localized mainly to the somas and apical dendritic processes of neuroreceptor cells.
Fox	Positive	Positive	Consistent labeling throughout the vomeronasal sensory neuroepithelium. The labeling pattern is similar to that in wolves with strong presence in the somas and apical regions.
Dog	Negative	Negative	No immunostaining observed in any part of the VNO, indicating a lack of V2R2 and Gαo expression.

## Data Availability

All relevant data are within the manuscript and are fully available without restriction.
